# Co-Immobilized Capillary Enzyme Reactor Based on Beta-Secretase1 and Acetylcholinesterase: A Model for Dual-Ligand Screening

**DOI:** 10.3389/fchem.2021.708374

**Published:** 2021-07-08

**Authors:** Adriana Ferreira Lopes Vilela, Vitor Eduardo Narciso dos Reis, Carmen Lúcia Cardoso

**Affiliations:** Departamento de Química, Grupo de Cromatografia de Bioafinidade e Produtos Naturais, Faculdade de Filosofia, Ciências e Letras de Ribeirão Preto, Universidade de São Paulo, Ribeirão Preto, Brazil

**Keywords:** co-immobilization procedure, dual enzymatic system assay, screening dual- target ligands, acetylcholinesterase, beta-secretase1

## Abstract

We have developed a dual enzymatic system assay involving liquid chromatography-mass spectrometry (LC–MS) to screen AChE and BACE1 ligands. A fused silica capillary (30 cm × 0.1 mm i.d. × 0.362 mm e.d.) was used as solid support. The co-immobilization procedure encompassed two steps and random immobilization. The resulting huAChE+BACE1-ICER/MS was characterized by using acetylcholine (ACh) and JMV2236 as substrates. The best conditions for the dual enzymatic system assay were evaluated and compared to the conditions of the individual enzymatic system assays. Analysis was performed in series for each enzyme. The kinetic parameters (K_Mapp_) and inhibition assays were evaluated. To validate the system, galantamine and a β-secretase inhibitor were employed as standard inhibitors, which confirmed that the developed screening assay was able to identify reference ligands and to provide quantitative parameters. The combination of these two enzymes in a single on-line system allowed possible multi-target inhibitors to be screened and identified. The innovative huAChE+BACE1-ICER/MS dual enzymatic system reported herein proved to be a reliable tool to identify and to characterize hit ligands for AChE and BACE1 in an enzymatic competitive environment. This innovative system assay involved lower costs; measured the product from enzymatic hydrolysis directly by MS; enabled immediate recovery of the enzymatic activity; showed specificity, selectivity, and sensitivity; and mimicked the cellular process.

## Introduction

Enzymes, play a fundamental role in cellular biochemical processes and are involved in many pathologies, have been investigated by researchers in the field of drug discovery and development. Investigations into the interaction of proteins and enzymes with target ligands provide essential information about biological systems, contributing to the design of new therapeutic interventions (Silber, 2010; Mignani et al., 2016; Cass et al., 2020).

Given that various small molecules are available for the production of new drugs and in view of the numerous biological targets known to date, new strategies must be adopted to develop relevant, reproducible, reliable, and robust moderate to high-throughput screening platforms for drug discovery (Janzen, 2014; Hage, 2017).

In this context, robust screening assays that employ enzymes immobilized on solid supports have received increasing attention because they offer advantages like improved stability and the possibility of proteins being reused (de Moraes et al., 2016). Such screening assays are based on bioaffinity chromatography, which combines the specificity and sensitivity of an enzymatic reaction with the automation and reproducibility of a chromatographic system (Acker and Auld, 2014; de Moraes et al., 2019).

Current approaches for high-throughput screening (HTS) are based on the search for compounds with efficacy toward a single target. However, a new paradigm is emerging in pharmacological research-exploration of the bioactivity of compounds at multiple targets (Mignani et al., 2016). This could enhance the therapeutic efficacy for diseases such as Alzheimer’s disease (AD), which has heterogeneous etiology and complex mechanism and is the most common form of dementia leading to neurodegeneration (Anand et al., 2014; Hughes et al., 2016; Ramsay et al., 2018). Indeed, existing treatments for AD have shortcomings or result in failure, so researchers have been devoted to changing drug design strategies to investigate multitarget-directed ligands (Hughes et al., 2016; de Freitas Silva et al., 2018; Maramai et al., 2020; Uddin et al., 2021).

Because the enzymes acetylcholinesterase and beta-secretase are the main targets involved in AD progression, they have been widely studied, paving the way for future pharmacological treatment of the disease (Deardorff et al., 2015; Coimbra et al., 2018). Acetylcholinesterase (AChE, EC 3.1.1.7), a carboxyl transferase found in neuromuscular junctions and cholinergic brain synapses, occurs in all species belonging to the animal kingdom. AChE regulates the end of the transmission of the neurotransmitter acetylcholine (ACh) in cholinergic synapses (Fotiou et al., 2015). In turn, beta-secretase 1 (BACE1, EC 3.4.23.48), an aspartyl protease found in the brain, plays a pivotal part in the formation of the myelin sheath in peripheral nerve cells. BACE1 initiates the proteolytic cleavage of the amyloid precursor protein (APP) at the luminal end of the cell membrane, forming the C-terminal fragment that is subsequently cleaved by γ-secretase at the N-terminal end, to generate the amyloid peptide (Aβ peptide) (Willem et al., 2006). Regulating both AChE and BACE1 during the treatment of AD is important and has motivated the scientific community to search for molecules that can inhibit these enzymes more efficiently (Deardorff et al., 2015; Fotiou et al., 2015; Prati et al., 2018).

Ligand screening studies based on assays with AChE (Vanzolini et al., 2018; Seidl et al., 2019) and BACE (Ferreira Lopes Vilela and Cardoso, 2017; Schejbal et al., 2019; Ye et al., 2021) immobilized on solid supports have been reported. However, the modulating effect of the screened bioactive compound may present limitations in the entire biological system *in vivo*, so targeting a single target can produce undesirable dual or synergistic effects (Pang et al., 2012). Therefore, developing dual enzymatic system assays that can mimic cellular process has become relevant for better understanding of these mechanisms of action in biological systems and drug discovery research.

Immobilized enzymes are widely used in many other sectors such as the pharmaceutical, chemical, and cosmetic industries, food processing, biofuel production, medical devices and biosensors, (Basso and Serban, 2019; Basso and Serban, 2020), and proteomic analyses by online protein digestion (Wang et al., 2016; Toth et al., 2017).

Multi-target systems can be developed by co-immobilization (Orrego et al., 2020) and have aroused interest due to their different applications, which include screening of ligands (Chen et al., 2017; Wu et al., 2020), syntheses of products (Betancor and Luckarift, 2010), design of biocatalyst cascades (Kang et al., 2014), and construction of biosensors to detect diseases (Zhang et al., 2012).

Given the inability of the one-target one-molecule strategy to develop efficient drugs for AD treatment over the last 20 years (Blaikie et al., 2019), we were motivated to develop a dual system assay to screen multitarget-directed ligands that are more fitting to AD treatment. To contribute to this field, here we report the development of an innovative, automated dual enzymatic system assay based on AChE and BACE1 co-immobilized on the inner surface of a fused silica capillary (30 cm × 0.1 mm i.d. × 0.362 mm e.d.) to screen ligands. Co-immobilization comprised two steps: first, the fused silica capillary was activated; then, the enzymes were covalently attached via amine-glutaraldehyde reaction. The resulting huAChE+BACE1-ICER was connected as a bioaffinity column to a liquid chromatographic system (LC) coupled to mass spectrometry (MS), to measure the activities of the enzymes. Comparisons with individual enzymatic systems were made. Next, the dual enzymatic system assay was validated, which allowed us to confirm standard inhibitors for both enzymes by determining the half-maximum inhibitory concentration (IC_50_). The dual enzymatic system assay proved to be an improvement on the HTS technique and can aid the search for multitarget-directed ligands in drug discovery for complex diseases.

## Materials and Methods

### Reagents and Solutions

The lyophilized powders of the enzymes β-secretase (BACE1, EC 3.4.23.48, human extracellular domain and recombinant C-terminal FLAG®-tagged expressed in HEK 293 cells) and acetylcholinesterase (huAChE EC 3.1.1.7, 1,000 units.mg^−1^ from human recombinant, expressed in HEK 293 cells), the β-secretase inhibitor, acetylcholine iodide (ACh), and galantamine hydrobromide were obtained from Sigma-Aldrich (St. Louis, MO, United States). The peptide substrate *ortho*-aminobenzoyl (Abz)-(Asn^670^, Leu^671^)-Amyloid β A4 Protein Precursor_770_ (669–674) – ethylenediamine 2,4-dinitrophenol (EDDnp) trifluoroacetate salt (named JMV2236 hereafter) was acquired from Bachem AG (Bubendorf, Switzerland). Glutaraldehyde solution (25%), dimethyl sulfoxide (DMSO), solution components, and all the chemicals used during the immobilization procedure were of analytical grade and were supplied by Sigma-Aldrich (St. Louis, United States), Merck (Darmstadt, Germany), Synth (São Paulo, Brazil), or Acros (Geel, Belgium).

The water used in all the preparations was deionized in a Millipore Milli-Q® system (Millipore, São Paulo, Brazil). The fused silica capillary (0.362 mm × 0.1 mm i.d.) was purchased from Polymicro Technologies (Phoenix, AZ, United States).

The mobile phases were prepared daily. The JMV2236 (1 mM) and β-secretase inhibitor (1 mM) stock solutions were prepared in DMSO and stored at -20°C. The ACh (1 mM) and galantamine (1 mM) stock solutions were prepared in ammonium acetate solution (15 mM, pH 4.5) and stored at -6°C.

### Instrumentation and System Configuration for Analyses

The LC-MS analyses were conducted on an LC system Nexera XR HPLC (Shimadzu, Kyoto, Japan) equipped with two LC 20AD pumps, an SIL-20A autosampler, a DGU 20A degasser, a CTO-20A oven, and an interfaced CBM-20A system. The LC system was coupled to an AmaZon speed Ion Trap (IT) Mass Spectrometry (MS) (Bruker Daltonics, Bremen, Germany) equipped with an Electrospray Ionization (ESI) interface. Individual and dual enzymatic systems (30 cm × 0.1 mm i.d. × 0.362 mm e.d.) were placed in the LC system as the bioaffinity column for on-flow MS.

The ESI ionization parameters were as follows: capillary voltage = 4.000 V, end plate voltage = 500 V, drying gas flow = 6 L min^−1^, drying temperature = 270°C, and nebulizer pressure = 18 psi. MS operating in the single ion-monitoring mode under positive ionization as follows: for huAChE (UltraScan 50–250 m*/z*), detection of the cations [M+H]^+^ *m/z* 146 (ACh) and [M+H]^+^ *m/z* 104 (Ch, the product of the enzymatic catalysis); for BACE1 (enhanced resolution 300–1,000 m*/z*), detection of the cations [M+H]^+^ *m/z* 987.5 (JMV2236) and [M+H]^+^ *m/z* 464.2 and *m/z* 542.1 (JMV2236 product of BACE1-catalyzed hydrolysis).

The LC analyses were performed at 37°C; 15 mM ammonium acetate solution pH 4.5 was used as mobile phase (pump A) at a flow rate of 20 μL min^−1^, and a second pump (B) delivered methanol after the ICER and before the IT-MS throughout a “T” shaped connection, at the same flow rate as the mobile phase of pump A (See Table 1).

**TABLE 1:** LC-MS conditions for measuring enzymatic activity in the huAChE+BACE1-ICER-LC-MS system.

LC-MS Conditions for dual and individual enzymatic assays		
	huAChE	BACE1
Substrate	ACh	JMV2236
Mobile phase	Pump A (isocratic mode): ammonium acetate solution, 15 mM, pH 4.5	
	Pump B (isocratic mode): methanol 100%	
Temperature	37°C	
Flow rate	0.02 ml min^−1^	
Injection volume	10 µL	
MS detection^a^	[M+H]^+^ *m/z* 104	[M+H]^+^ *m/z* 464.2
		[M+H]^+^ *m/z* 542.1
Stop flow	0 min	5 min
Analysis time	5 min	10 min

a mean error ± 0.5.

The data were collected with the Bruker Daltonics Data Analysis software (version 4.3).

A syringe pump model 11 Plus advanced single (Harvard Apparatus, Holliston, United States) was used to infuse all the solutions during the immobilization procedure.

### Preparation and Evaluation of Individual Enzymatic Systems

#### huAChE or BACE1 Immobilization

huAChE (17 units.ml^−1^ in 50 mM phosphate solution, pH 7.4) and BACE1 (942.8 units.ml^−1^ in 50 mM phosphate solution, pH 7.4) were immobilized, separately, on the internal surface of an open tubular silica capillary (100 cm × 0.1 mm i.d × 0.362 mm e.d.) by following previous protocols for individual enzyme immobilization as described in (Ferreira Lopes Vilela and Cardoso, 2017).

In each case, the 100-cm capillary was divided, to produce three 30-cm-long huAChE-ICERs or BACE1-ICERs.

#### Influence of the pH of the LC-MS System Mobile Phase

For huAChE-ICER and BACE1-ICER, 10-µL aliquots of ACh (70 µM) or JMV2236 (100 µM) solution at different pH values (from 4.0 to 6.0) were injected into the individual enzymatic system operating at 0.02 ml min^−1^ and 37°C.

#### Apparent Kinetic Constant (K_Mapp_) Studies

The K_Mapp_ constant of huAChE-ICER was obtained by using different ACh concentrations (25, 50, 75, 100, 125, 150, 250, or 1,000 μM) in ammonium acetate solution (15 mM, pH 4.5). To this end, 20 μL of each solution was further diluted to a final volume of 100 μL with ammonium acetate solution (15 mM, pH 8.0 or pH 4.5). The final solutions were vortex-mixed for 10 s, and 10-μL aliquots were injected into the huAChE-ICER-LC-MS system in triplicate.

The K_Mapp_ constant of BACE1-ICER was obtained. Briefly, 5-µL aliquots of JMV2236 solution at various concentrations (from 10 to 100 µM) were injected into the BACE1-ICER-MS system, in triplicate. The assayed solutions were obtained by addition of 25 µL of JMV2236 solution at different concentrations, completed to a final volume of 50 µL with ammonium acetate solution (2 mM, pH 4.5) as described in Ferreira Lopes Vilela and Cardoso (2017).

The data were fitted by using nonlinear regression in a Michaelis-Menten plot, and the K_Mapp_ values were obtained with the software GraphPad Prism version 5.0.

#### Inhibition Studies

To determine the half-maximum inhibitory concentration (IC_50_), galantamine and a β-secretase inhibitor were used as reference inhibitors for BACE1-ICER and huAChE-ICER, respectively (Andrau et al., 2003; Darvesh et al., 2003; Marques et al., 2011).

For huAChE-ICER, 10-µL aliquots of solutions containing increasing galantamine (0.05–50 µM) concentrations and ACh (70 µM) were injected into the huAChE-ICER-LC-MS system, in triplicate. The assay solutions were obtained by using 20 µL of a solution containing ACh (350 µM) and 10 µL of a solution containing different galantamine concentrations (0.05, 1.0, 2.5, 5.0, 7.0, 10, 15, 25, or 50 µM). The final volume was completed to 100 µL with ammonium acetate solution (15 mM, pH 4.5).

The product areas obtained in the presence (Pi) and absence (P0) of the inhibitor were compared, and the percentage of inhibition was calculated by using Eq. 1:

$$\%\;Inhibition=100-\left[\left(\frac{Pi}{P0}\right)\;x\;100\right]$$ (1)

For each standard inhibitor, an inhibition curve was constructed by plotting the % inhibition versus the concentration of the inhibitor. The software GraphPad Prism 5 was used to obtain the IC_50_ values by nonlinear regression analysis.

The IC_50_ for BACE1 was obtained by injecting 5 uL aliquots of solutions containing increasing concentrations of the β-secretase inhibitor (from 0.05 to 10 µM) and JMV2236 (5 µM) into the BACE1-ICER-MS system, in triplicate. The assayed solutions were obtained by addition of 10 µL of JMV2236 (50 µM) and 10 µL of a solution containing different concentrations of reference inhibitor, completed to a final volume of 50 µL with ammonium acetate solution (2 mM, pH 4.5) (Ferreira Lopes Vilela and Cardoso, 2017).

### Preparation and Evaluation of the Dual Enzymatic System

#### huAChE and BACE1co-Immobilization

huAChE and BACE1 were co-immobilized on the internal surface of an open tubular silica capillary (100 cm × 0.1 mm i.d. × 0.362 mm e.d.) according to previous protocols described for individual enzymes (Vanzolini et al., 2013; Ferreira Lopes Vilela and Cardoso, 2017), with minor modifications.

A mixture of the enzymes was infused through the capillary at 50 μL min^−1^. The mixture consisted of 500 μL of huAChE solution (17 units.ml^−1^ in 50 mM phosphate solution, pH 7.4) and 500 μL of BACE1 solution at 942.8 units.ml^−1^ (20 µL of BACE1 solution in 20 mM Hepes solution, pH 7.4, containing 125 mM NaCl, diluted to 500 ml with 50 mM phosphate solution, pH 7.4). The solution was collected, and this step was repeated five times by using the same solution.

The 100-cm capillary was divided, to give three 30-cm-long huAChE+BACE1-ICERs.

While huAChE+BACE1-ICER was not being used, it was maintained at 4°C, with both extremities immersed in 50 mM phosphate solution, pH 7.4.

#### Apparent Kinetic Constant (KMapp) Studies

The apparent kinetic constant (KMapp) was obtained independently for both substrates, ACh and JMV2236, by varying their concentrations.

For the huAChE, ACh stock solutions were prepared (25, 50, 75, 100, 125, 150, 250, and 1,000 μM) in ammonium acetate solution (15 mM, pH 4.5), and 20 μL of each solution was further diluted to a final volume of 100 μL with the ammonium acetate solution (15 mM, pH 4.5). The final solutions were vortex-mixed for 10 s, and 10-μL aliquots were injected into the huAChE+BACE1-ICER-LC-MS system, in triplicate.

For the BACE1, JMV2236 stock solutions were prepared (40, 60, 80, 100, 160, 200, 400, and 1,000 µM) in DMSO, and 25 µL of each solution was completed to a final volume of 50 µL with ammonium acetate solution (15 mM, pH 4.5). The final solutions were vortex-mixed for 10 s, and 10-μL aliquots were injected into the huAChE+BACE1-ICER-LC-MS system, in triplicate. The conditions are listed in Table 1.

The software GraphPad Prism 5 was used to obtain Michaelis–Menten plots by nonlinear regression analysis.

#### 3) Inhibition Studies

To validate the assay, IC_50_ parameters were calculated for β-secretase inhibitor and galantamine, standard inhibitors for huAChE and BACE1, respectively (Andrau et al., 2003; Darvesh et al., 2003).

For each enzyme, fixed concentrations of their substrates with increasing concentrations of the standard inhibitors were employed.

For the huAChE, 10-µL aliquots of solutions containing increasing galantamine concentrations (0.05–50 µM) and ACh (70 µM) were injected into the huAChE+BACE1-ICER-LC-MS system, in triplicate.

The assay solutions were obtained by adding 20 µL of a solution containing ACh (350 µM) and 10 µL of a solution containing different galantamine concentrations (0.05, 1.0, 2.5, 5.0, 7.0, 10, 15, 25, or 50 µM). The final volume was completed to 100 µL with ammonium acetate solution (15 mM, pH 4.5).

For the BACE1, 10-µL aliquots of solutions containing increasing concentrations of the β-secretase inhibitor (0.05–10 µM) and JMV2236 (100 µM) were injected into the huAChE+BACE1-ICER-LC-MS system, in triplicate.

The assay solutions were obtained by adding 10 µL of a solution containing JMV2236 (1,000 µM in DMSO) and 10 µL of a solution containing different concentrations of the β-secretase inhibitor (0.05, 0.1, 0.25, 0.5, or 2.5 in DMSO). The final volume was completed to 50 µL with ammonium acetate solution (15 mM, pH 4.5).

The assay conditions are described in Table 1.

The percentage of inhibition was calculated according to Eq. 1.

For each standard inhibitor, an inhibition curve was constructed by plotting the % inhibition versus the concentration of the inhibitor. The software GraphPad Prism 5 was used to obtain the IC_50_ values by nonlinear regression analysis.

## Results and Discussion

### Individual Enzymatic System Assay

Before we developed the dual enzymatic system assay by co-immobilization of huAChE and BACE1, we studied the individual enzymes huAChE and BACE1 on the basis of previous works by Vanzolini et al. (2013), Ferreira Lopes Vilela and Cardoso (2017) to evaluate the best conditions for monitoring both enzymes in the dual enzymatic system assay by LC-MS.

We successfully immobilized huAChE and BACE1, individually, on the internal surface of an open tubular fused silica capillary, to obtain huAChE-ICER and BACE1-ICER, respectively.

The pH study was crucial because the enzymes have optimal catalytic functions at different pH levels. BACE1 is an aspartyl protease with optimal activity in acidic pH, and AChE, an esterase, works best at pH between 7 and 8.5. On the basis of this information, we investigated the ideal pH for the dual enzymatic system assay.

The enzymatic activity can also be determined at a pH outside the optimum range, but smaller activity values must be accepted. In this way, a larger number of enzymes can be measured at one standardized pH (Bisswanger, 2014).

**FIGURE 1:**
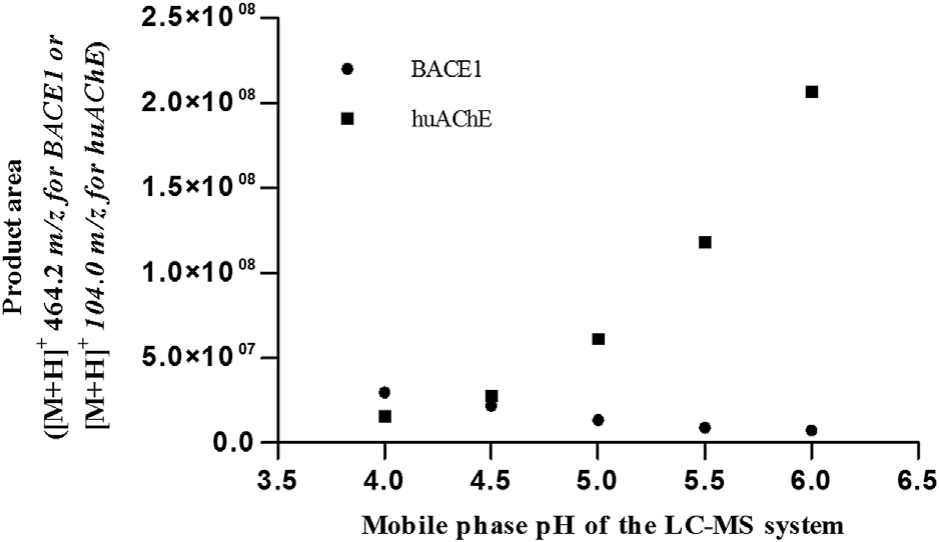
Activities of huAChE-ICER and BACE1-ICER in individual enzymatic system assays as a function of the pH of the mobile phase.

**FIGURE 2:**
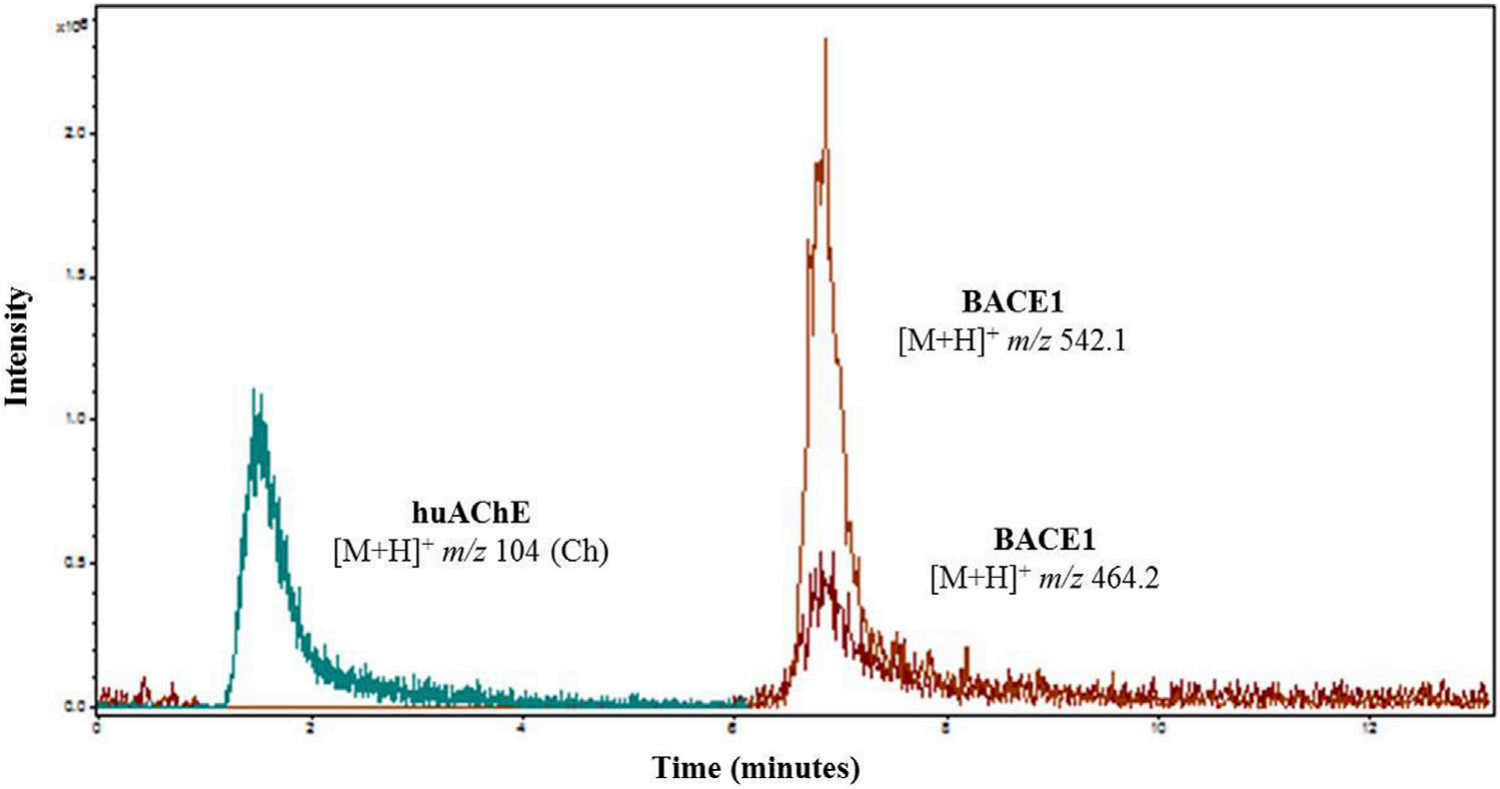
Overlap of extracted ion chromatogram obtained after injection of 50 µM ACh into the huAChE-ICER-LC-MS system and 50 µM JMV2236 into the BACE1-ICER-LC-MS system. The products of enzymatic hydrolysis were monitored at pH 4.5.

According to Figures 1, 2, at pH values above 5.5, BACE1-ICER does not function well. As for huAChE-ICER, we were able to evaluate its activity in a wide pH range. Our results showed that the ideal pH of the mobile phase for monitoring the dual enzymatic system should be 4.5.

On the basis of these studies, we standardized the mobile phase pH so as to measure the enzymatic catalysis for both enzymes in the dual enzymatic system assay.

The constant K_Mapp_ depends on the reaction medium conditions, so we investigated changes in this constant as a function of the conditions of the enzymatic reactions. BACE1-ICER had been previously studied, and its K_Mapp_ is described in Ferreira Lopes Vilela and Cardoso (2017). As for huAChE-ICER, K_Mapp_ studies were carried out for two mobile phase pH values, 8.0 (optimal for huAChE) and 4.5 (selected for monitoring of the dual enzymatic system assay).

**FIGURE 3:**
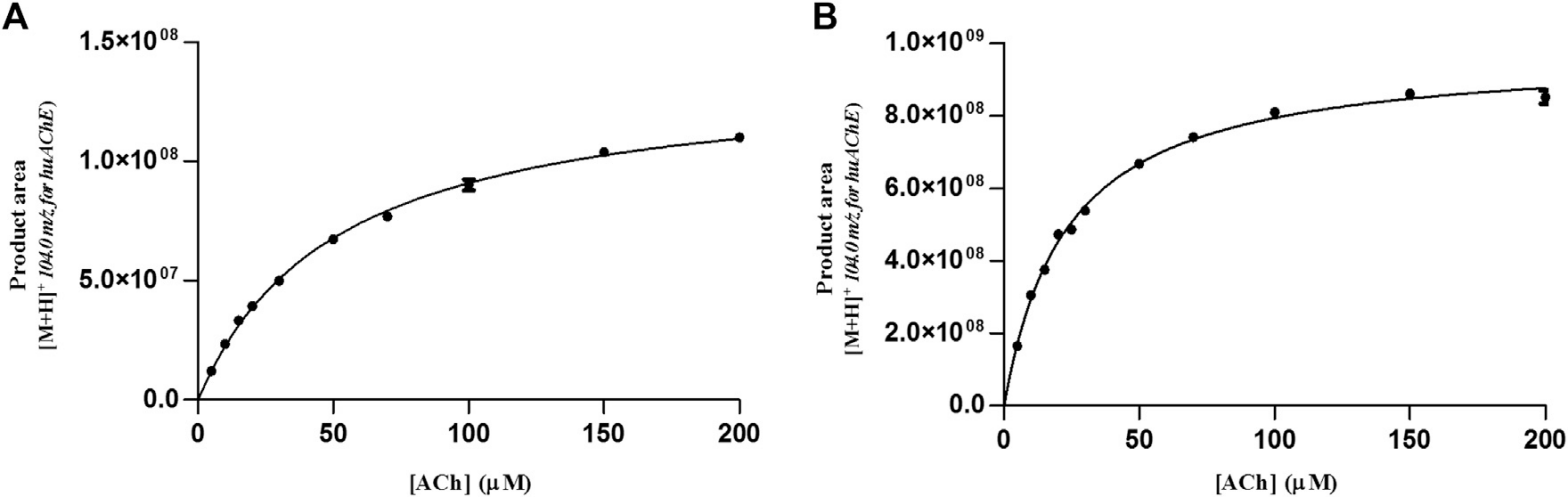
Michaelis-plots of the product area obtained for huAChE-ICER by using ACh as the substrate. **(A)** mobile phase pH 4.5 and **(B)** mobile phase pH 8.0. See Table 1 for conditions.

K_Mapp_ was calculated as 51.5 ± 2 µM at pH 4.5 and 23.3 ± 1 µM at pH 8.0 (Figure 3). Compared to the value obtained at the optimal pH (8.0) for huAChE, K_Mapp_ at pH 4.5 was twice as large and will be used to carry out the ligand screening studies.

To validate the huAChE-ICER-LC-MS system for ligand screening, we used galantamine, a known inhibitor of cholinesterases (Darvesh et al., 2003). We also calculated the IC_50_ values for huAChE-ICER at two mobile phase pH values, 8.0 (optimal for huAChE) and 4.5 (selected for monitoring of the dual enzymatic system assay). The IC_50_ value for BACE1 has been described in (Ferreira Lopes Vilela and Cardoso, 2017).

**FIGURE 4:**
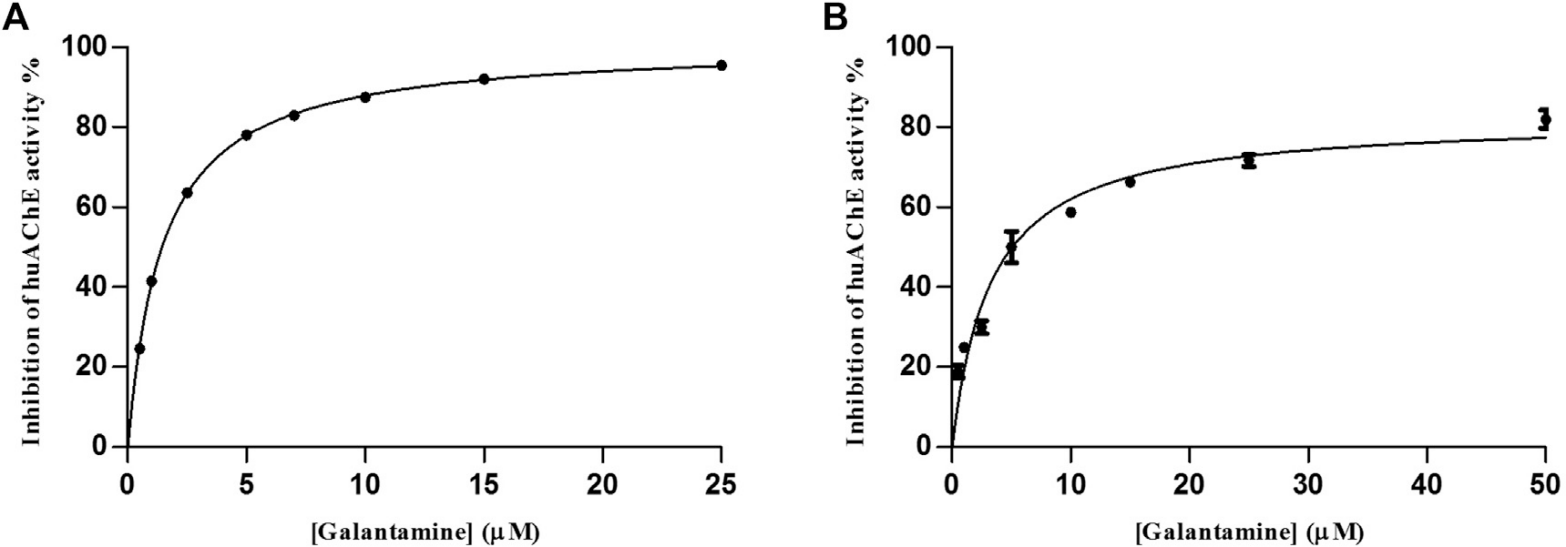
Inhibitory potency curve obtained for huAChE-ICER by using ACh as a substrate. **(A)** mobile phase pH 4.5 and **(B)** mobile phase pH 8.0. See Table 1 for conditions.

We determined the IC_50_ values as 3.29 ± 0.4 µM at pH 4.5 and 1.47 ± 0.03 µM at pH 8.0 (Figure 4), which showed that the huAChE-ICER-LC-MS system was able to identify the standard inhibitor on a micro-scale as compared to data previously reported for this inhibitor.

On the basis of these findings for the individual enzymatic system assays, we were able to establish the best conditions for the dual enzymatic system assay (Table 1).

### Dual Enzymatic System Assay

**FIGURE 5:**
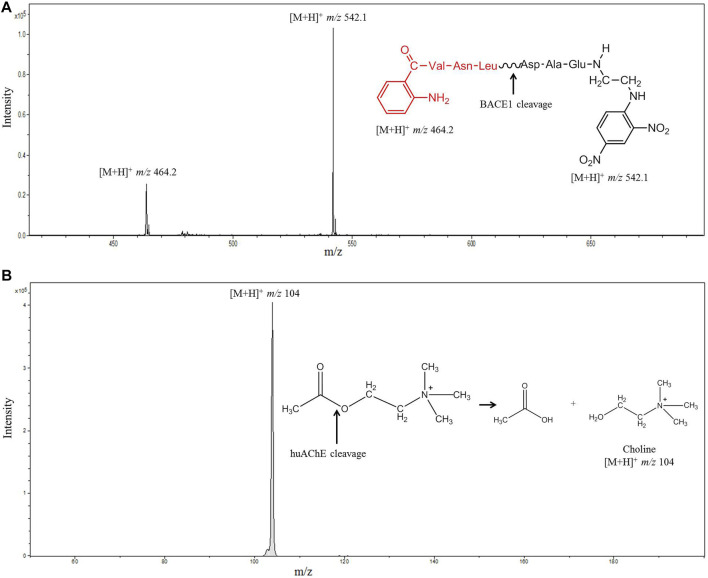
Mass spectrum obtained after injection of JMV2236 (100 µM) into the huAChE+BACE1-ICER-LC-MS system. **(A)** The product of enzymatic hydrolysis ([M+H]^+^ *m/z* 464.2 and 542.1) was monitored. **(B)** Mass spectrum obtained after injection of ACh (70 µM) into the huAChE+BACE1-ICER-LC-MS system by monitoring the product of enzymatic hydrolysis ([M+H]^+^ *m/z* 104) (Conditions listed in Table 1).

The co-immobilization process used herein was successful: the activities of both enzymes were preserved after co-immobilization in the fused silica capillary. The random co-immobilization method (Schoffelen and van Hest, 2013) employed glutaraldehyde as linker, to produce covalent interactions with the mixture of enzymes on the support. The LC-MS system and the configuration used to monitor the enzymatic activities allowed us to measure the hydrolysis products by MS directly. We analyzed both enzymes in series, BACE1 and huAChE, respectively. Figure 5 illustrates the results.

We validated the assay according to guidelines for bioanalytical methods (Cassiano et al., 2009). The calibration curve revealed a linear relationship for JMV2236 concentrations ranging from 10 to 25 µM. For ACh, the calibration curve was linear (1.0–15 μM).

To evaluate the selectivity of the method, we carried out blank runs (15 mM ammonium acetate pH 4.5), where the target analytes were not present. These runs revealed no interference, demonstrating that no carry over occurred between analyses.

For ACh, the limit of quantification (LQ) was 1 µM (RSD = 2.4%, *n* = 3, and accuracy = 100.0%), and the limit of detection (LD) was 0.5 µM, calculated by the signal-to-noize ratio. For JMV2236, the limit of quantification (LQ) was 10 µM (RSD = 9.2%, *n* = 3, and accuracy = 100.6%), and the limit of detection (LD) was 5.0 µM, calculated by the signal-to-noize ratio.

**FIGURE 6:**
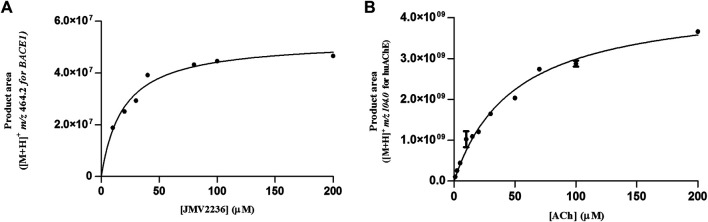
Michaelis-Menten plots of the product area obtained for huAChE **(A)** and BACE1 **(B)** in the huAChE+BACE1-ICER-LC-MS system by using ACh and JMV2236 as substrates, respectively.

We evaluated the K_Mapp_ constants for both enzymes in the dual enzymatic system, to verify whether the values changed due to co-immobilization. We found values of 19.25 ± 3 and 49.6 ± 4 µM for BACE1 and huAChE in the huAChE+BACE1-ICER-LC-MS system, respectively (Figure 6).

Table 2 compares the K_Mapp_ values obtained for the dual and individual system assays. There were no significant differences due to the random co-immobilization and immobilization process.

**TABLE 2:** K_Mapp_ values obtained for the dual and individual system assays.

K_Mapp_ values (µM)	*huAChE*			*BACE1*		
	huAChE*-ICER* ^a^	huAChE+BACE1-ICER^b^	Ratio^c^	BACE1-ICER^a^ ^,^ ^d^	huAChE+BACE1-ICER^b^	Ratio^c^
	51.5	49.6	0.96	17.2	19.2	1.1

a Individual-system.

b Dual-system.

c Ratio between dual and individual-system.

d Value described in Ferreira Lopes Vilela and Cardoso (2017).

The individual and dual enzymatic systems developed herein showed that the enzymes catalyzed their substrates in a similar fashion in both systems; that is, their affinities and specificities were not affected. Therefore, we were able to mimic an *in vitro* cellular environment with two enzymes with distinct catalytic properties in a unique system. Other advantages of the proposed method include reduced costs of reagents and shorter preparation time. In addition, the same mobile phase and bioaffinity column can be used in the analyses, allowing ligands from synthetic or semi-synthetic combinatorial libraries and natural products or marine extracts to be identified and characterized, not to mention that the method can be applied to multi-target ligands. The proposed dual-enzymatic system assay did not allow the two substrates and products cleaved by the enzymes to be detected simultaneously due to the employed MS detector. However, the biocatalysis products were directly quantified in series by an MS detector.

**FIGURE 7:**
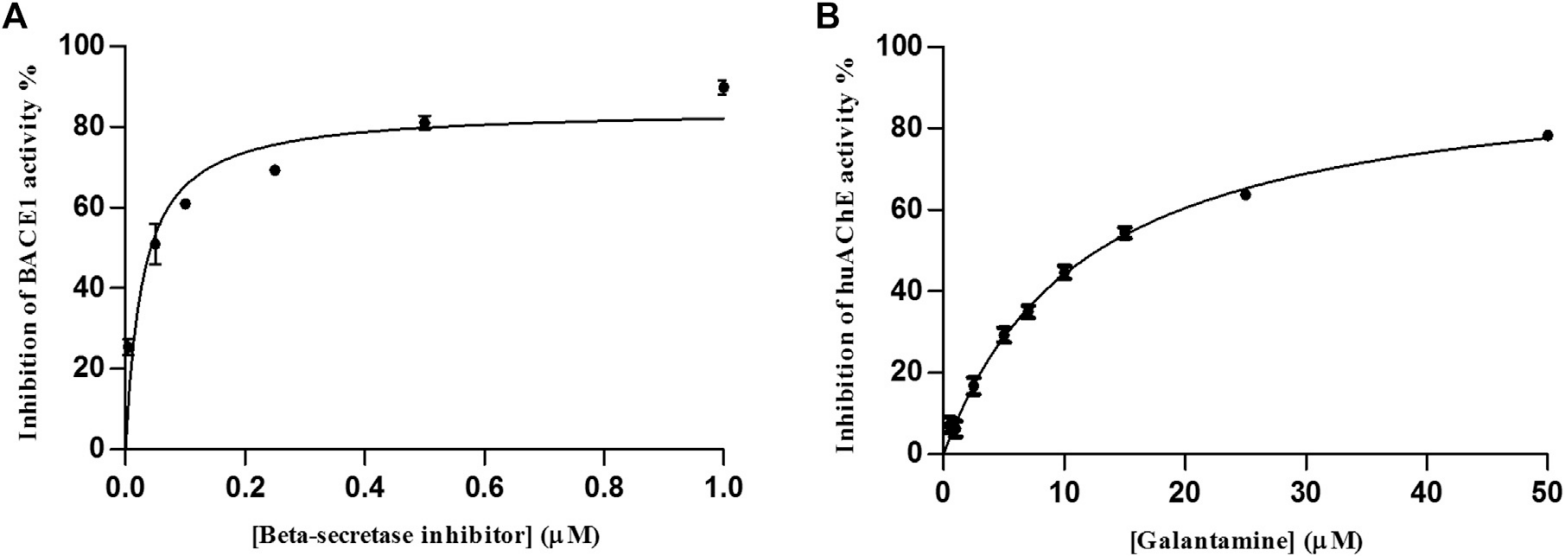
IC_50_ curve obtained for huAChE **(A)** and BACE1 **(B)** in the huAChE+BACE1-ICER-LC-MS system by using ACh and JMV2236 as substrates, respectively.

Although the assay for huAChE was not performed under optimal catalysis conditions, we were able to evaluate its activity and selectivity with the same efficacy provided by individual enzymatic system. To validate the huAChE+BACE1-ICER-LC-MS system for the screening of ligands, we used galantamine and the β-secretase inhibitor as reference inhibitors of BACE-1 and huAChE, respectively (Andrau et al., 2003; Darvesh et al., 2003). The IC_50_ values were 11.6 ± 0.8 µM for galantamine and 0.03 ± 0.008 µM for the β-secretase inhibitor (Figure 7). On the basis of these values, the dual enzymatic system assay was able to identify the standard inhibitors on a micro-scale as compared to data previously reported for these inhibitors.

Each enzyme studied in the dual enzymatic system was selective and specific for its substrate and inhibitor without interference and/or problems during method development (30 days); the enzymatic activities were maintained for over 60 days. The K_Mapp_ and IC_50_ studies showed that co-immobilization did not alter the affinity of BACE1 and huAChEhu, indicating that the approach used herein can be applied to screen multi-target-directed ligands in a competitive environment. This efficient system is a remarkable alternative to common single-enzyme screening methods at various levels during the drug discovery process.

## Conclusion

We have described an innovative dual enzymatic system assay based on co-immobilization of huAChE and BACE1 and on-flow LC-MS detection. This is the first time that a dual enzymatic system assay has been developed to screen ligands by a label-free automated screening system bearing two enzymes involved in AD etiology. We developed the novel dual enzymatic system assay by employing an effective random co-immobilization process that provided a system to evaluate the activities of huAChE and BACE1 on-flow by LC-MS through a mobile phase with the same ionic strength and pH even though the enzymes have different kinetic properties.

## Data Availability

The raw data supporting the conclusions of this article will be made available by the authors, without undue reservation.
